# Molecular Approaches to Overcome Self-Incompatibility in Diploid Potatoes

**DOI:** 10.3390/plants11101328

**Published:** 2022-05-17

**Authors:** Hemant Balasaheb Kardile, Solomon Yilma, Vidyasagar Sathuvalli

**Affiliations:** 1 Department of Crop and Soil Science, 109 Crop Science Building, Oregon State University, Corvallis, OR 97331, USA; kardileh@oregonstate.edu (H.B.K.); solomon.yilma@oregonstate.edu (S.Y.); 2 Division of Crop Improvement and Seed Technology, ICAR-Central Potato Research Institute, Shimla 171001, Himachal Pradesh, India; 3 Hermiston Agricultural Research, and Extension Center, Hermiston, Department of Crop and Soil Science, Oregon State University, Hermiston, 2121 South 1st Street, Hermiston, OR 97838, USA

**Keywords:** F_1_ hybrid, S-RNAse, S-locus, *S-locus inhibitor (Sli)*

## Abstract

There has been an increased interest in true potato seeds (TPS) as planting material because of their advantages over seed tubers. TPS produced from a tetraploid heterozygous bi-parental population produces non-uniform segregating progenies, which have had limited uniformity in yield and quality in commercial cultivation, and, thus, limited success. Inbreeding depression and self-incompatibility hamper the development of inbred lines in both tetraploid and diploid potatoes, impeding hybrid development efforts. Diploid potatoes have gametophytic self-incompatibility (SI) controlled by S-locus, harboring the male-dependent *S-locus F-box* (*SLF/SFB)* and female-dependent *Stylar-RNase (S-RNase).* Manipulation of these genes using biotechnological tools may lead to loss of self-incompatibility. Self-compatibility can also be achieved by the introgression of *S-locus inhibitor* (*Sli*) found in the self-compatible (SC) natural mutants of *Solanum chacoense*. The introgression of *Sli* through conventional breeding methods has gained much success. Recently, the *Sli* gene has been cloned from diverse SC diploid potato lines. It is expressed gametophytically and can overcome the SI in different diploid potato genotypes through conventional breeding or transgenic approaches. Interestingly, it has a 533 bp insertion in its promoter elements, a MITE transposon, making it a SC allele. *Sli* gene encodes an F-box protein PP2-B10, which consists of an F-box domain linked to a lectin domain. Interaction studies have revealed that the C-terminal region of Sli interacts with most of the StS-RNases, except StS-RNase 3, 9, 10, and 13, while full-length *Sli* cannot interact with StS-RNase 3, 9, 11, 13, and 14. Thus, *Sli* may play an essential role in mediating the interactions between pollen and stigma and function like SLFs to interact with and detoxify the S-RNases during pollen tube elongation to confer SC to SI lines. These advancements have opened new avenues in the diploid potato hybrid.

## 1. Introduction

The potato (*Solanum tuberosum* L.) is an important food crop. Tubers are the planting material in commercial cultivation. Several problems are associated with tuber-based cultivation. Among them, their high cost, need for disease-free high-quality tubers and seed-tuber storage costs affect economic gain. Interest in using botanical true potato seeds (TPS) as planting material has recently increased because of its advantages over tubers. TPS-based cultivation would be free from tuber-borne diseases and reduce logistic expenditures for storage and transportation [1]. To date, TPS has been applied to varietal development, wherein the TPS of bi-parental crosses are used for genetic progress and clonal selection, as TPS are highly heterozygous and segregate for various traits.

**Figure 1:**
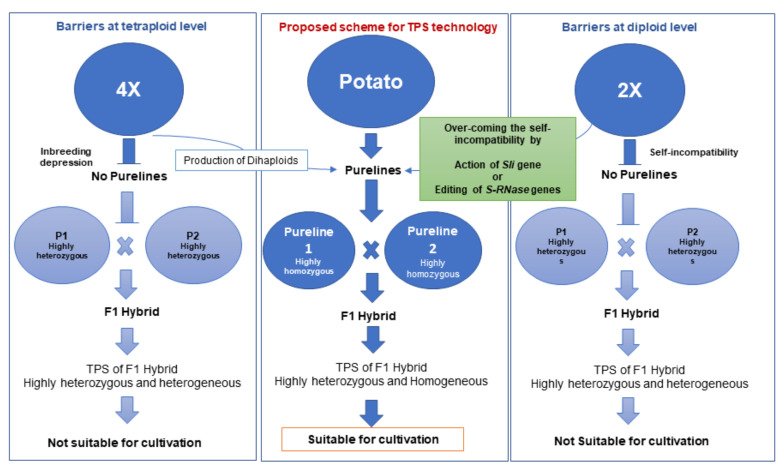
Molecular approaches to overcome self-incompatibility in diploid potatoes. Self-incompatibility barriers at the diploid level can be overcome by the action of the *Sli* gene or by editing the S-locus (*S-RNase*) gene.

The level of heterozygosity and heterogeneity within the family increases with each generation; hence, TPS have limited application in commercial potato cultivation [2]. TPS would be of greater benefit to commercial potato cultivation if its use resulted in genetically uniform progenies. Relatively homozygous parents could make this possible. Due to the expression of deleterious alleles, self-fertilization in autotetraploid potatoes causes severe inbreeding depression. Little success was achieved when the homozygous lines were created from anther and ovule culture. This is no longer practiced because of technical difficulties, low yields, and poor fertility [2]. For this reason, breeders have begun generating inbred lines at the diploid level rather than at the tetraploid level. Further, most of the diploid (2*n* = 2*x* = 24) tuber-bearing potato species are self-incompatible (SI) [3]. This limits the value of diploids in generating such valuable genetic resources as inbred and recombinant inbred lines. Challenges associated with self-incompatibility and inbreeding at the diploid and tetraploid level and possible ways to overcome these are summarized in Figure 1 and explained in greater detail in the review. The purpose of this review is to document the key events in the progress of the development of the diploid F_1_ hybrid. With the recent discovery of the gene responsible for overcoming self-incompatibility in diploid potatoes, this review provides details about applying molecular tools to overcome self-incompatibility.

## 2. Molecular Basis of Self-Incompatibility (SI)

Most flowering plants have hermaphroditic flowers with proximate reproductive organs; this feature has permitted self-pollination and self-fertilization in most angiosperms. However, self-fertilization leads to poor genetic diversity and inbreeding depression, so nature has evolved the SI mechanism to prevent the deleterious effects of self-fertilization [4]. SI is a prezygotic barrier that prevents self-fertilization. The pistil differentiates self-pollen from non-self-pollen and prevents fertilization by self-pollen [5]. Based on genetic determinants, SI is of two types: gametophytic and sporophytic self-incompatibility [6]. Gametophytic self-incompatibility (GSI) is widespread and found in more than 60 flowering plant families, including the Solanaceae [4,7]. Extensive studies on GSI reveal two different molecular mechanisms, stylar ribonuclease (S-RNase) and pollen receptor mechanisms, that enable the pistil to identify and reject self-pollen. S-RNase-based GSI is controlled by a single, highly polymorphic S-locus, encoding for style-specific S-RNase genes and several pollen-specific *S-locus F-box* (*SLF/SFB*) genes. These F-box proteins are involved in ubiquitin-mediated protein degradation, which uses a cascade of E1, E2, and E3 enzymes to form polyubiquitin chains on specific substrates for degradation by the 26S proteasome. E1 enzymes are ubiquitin-activating enzymes; E2 are ubiquitin-conjugating enzymes, and the E3 enzyme is a ubiquitin ligase [8]. Molecularly, SI is due to the ribonuclease activity of *S-RNases* inhibiting pollen tube extension in the style [9,10], whereas self-compatibility (SC) is the result of ubiquitination of S-RNases mediated by the pollen determinant *SLF/SFB* [11,12]. S-RNase is a polymorphic glycoprotein, ribonucleases (S-RNases) with confirmed RNA degradation activity. Transgenic experiments in petunia and tobacco have established that S-RNase is the sole determinant of pistil specificity [9,10]. It was initially identified and characterized in members of the Solanaceae family, and later in the Rosaceae, Plantaginaceae, and most recently in the Rubiaceae [13,14,15]. The collaborative non-self-recognition model predicted that each *SLF*’s allelic variant might cause ubiquitin-mediated degradation of some of its non-self-stylar RNases [16]. The combined action of the male determinant *SLFs* of one S-haplotype facilitates ubiquitinoylation of all enzymes except its S-RNases [17]. Thus, SI prevails when the pollen’s S-haplotype matches either of the two S-haplotypes from the stylar portion. Papaveraceae family members have another SI mechanism. This involves the stigmatic *S-gene*, which presumably acts as a signal molecule, triggering the cascade of signal transduction events in incompatible pollen tubes, leading to inhibition of pollen tube growth. In Papaveraceae, SI appears to function differently than Solanaceae but may have important implications for the evolutionary relationships between the *S-genes* [18,19]. Although general in nature, self-incompatibility is distributed unevenly in potato germplasm.

## 3. Self-Incompatibility in Potatoes

Most of the diploid tuber-bearing Solanum species have a GSI. A single multi-allelic S-locus controls self-incompatibility in the diploid potatoes [3,20], located on chromosome 1 [21,22]. The multi-allelic S-locus undergoes few recombination events, maintaining its two genes on the same locus and bringing out the SI. [12]. Interestingly, some tetraploids are self-compatible because of heteroallelic diploid pollen, which has two different sets of SLFs, enabling mutual weakening or competitive interaction [16] and leading to the degradation of all S-RNases. Different Solanum species, mostly diploids, have natural mutants for self-compatibility; for example, *S. chacoense* clone chc 525-3 was the first to be reported. *S. chacoense* is otherwise self-incompatible. The presence of *Sli* may be responsible for the underlying molecular mechanism of self-compatibility observed in this clone [23]. Some other prominent clones reported to be self-compatible are *S. stenotomum* landrace Huasa Amarilla (C151 or CIP 705468) [24], M6 (originally, chc 523-3) [2], *Solanum tuberosum* diploid, RH89-039-16 [25] and two dihaploids, US-W4 [26] and G254 [27]. SC clones from different potato species that have been used to elucidate SI in diploid potatoes are given in Table 1. Other SC clones may be present in the large, diverse potato germplasm; these may provide even greater insight into SI. However, the identification of a SC phenotype is complex and shows quantitative rather than qualitative variation, which may make their identification challenging. The availability of SC markers, cloning of the gene providing SC (*Sli*), and robust phenotyping methods have eased the task of screening large sets of germplasm for SC. Mapping of SC QTLs, marker development, and cloning of *Sli* gene in potatoes are discussed in greater detail below.

## 4. Phenotyping for Self-Compatibility (SC)

Initially, controlled self-pollination followed by a berry and seed set was the only parameter used to identify SC in potatoes [23]. However, there is a need for comprehensive phenotyping of many reproduction-related traits to study SC in diploid potatoes, as berries set upon selfing result from self-fertility rather than self-compatibility [25]. The absence of berry formation after selfing can be due to self-incompatibility as well as infertility of male or female gametes, lack of gamete fusion, and embryo abortion [35]. Spontaneous berry development is a rare phenomenon in the diploid potato. Self-compatible diploids are rare, with SI being seen as the primary reason.

In practice, the potato is vegetatively propagated; this results in low selection pressure for fertility traits. Compared to their tetraploid relatives, ploidy reduction in diploids exposed deleterious recessive mutations that further aggravate fertility problems. Hence, phenotyping other reproductive-related characteristics such as pollen fertility and pollen tube growth after selfing would assess the potato’s SC status. Degradation of S-RNases allows the development of pollen tubes to carry out self-fertilization. This mechanism can be studied 48 h after self-pollination and has provided a new phenotyping tool to distinguish SI from SC more clearly than indirect or confounding markers like a berry set. Different stains, such as aniline blue fluorochrome [36] and DAPI under a UV microscope [30], permit the study of pollen tube growth within the style. However, this phenotypic trait shows quantitative variation, which has been classified into four classes: no pollen tube, few pollen tubes (<20), some pollen tubes, and many pollen tubes. An arbitrary scale of 0–3 for these possibilities is used, with 0 assigned to no pollen tubes and 3 to many pollen tubes. 

Based on a previous study [30], the recommendation is to record both pollen tube growth and berry set to unambiguously identify the diploid SC and SI plants. For example, clones with a 75% berry set and a substantial number of pollen tubes reaching the ovaries may have been classified as SC [30]. Over time, a more stringent and rigorous SC phenotyping protocol might include berry and seed set from both the cross and self-pollination and visualization of pollen tube growth in the style to avoid sterility confounding the compatibility phenotype. Plants that set more than one self-berry are considered SC, whereas plants that do not set self-berries after at least 10 self-pollinations, show self-pollen tube growth arrest in style, or set cross-berries after pollination with bulked pollen are considered SI. Improved extensive phenotyping permits screening of a large set of potato germplasm for self-compatibility. This approach has identified a new SC source, which has provided a means to investigate the underlying S-locus and S-locus inhibitor more comprehensively. Though robust SC phenotyping is in place, it is labor-intensive and shows quantitative variation. As a result, there is a pressing need to establish molecular markers associated with SC, which necessitates a thorough analysis of SI governing genes.

## 5. Molecular Analysis of the Essential Genes of SI

Female determinant *S-RNase* and male determinant *SLF/SFB* are the key genes governing SI in potatoes. Different potato species, mostly diploids, have been reported to contain a total of 49 *S-RNase* sequences [33,36,37]. Allelic variants of *S-RNase* show gametophytic control over pollen tube growth, wherein matching alleles are efficiently rejected after pollination and prevented from appearing in zygotes. Plants exhibiting the *S-RNase*-based GSI system are expected to be heterozygotes bearing two different S-alleles, suggesting the co-dominate nature of S-RNase [36].

Primary structural features of solanaceous S-RNases include three conserved domains (C3–C5) and two hypervariable domains (HVa and HVb). RNA degradation activity of S-RNAse is due to two catalytic histidine residues (His). Sequence alignment has shown that His residues are present in conserved C2 and C3 regions of this protein. A variable number of potential N-glycosylation sites are present in S-RNase. However, there exists one conserved potential N-glycosylation site in the C2 conserved region. This site could be responsible for modulating the ribonuclease activity of S-RNase [37,38]. Studies indicate that removal of the glycan side chain in these sequences neither altered the enzymatic activity of the *S-RNase* gene in vitro [39] nor its function in self-incompatibility in transgenic *Petunia inflata* [40]. With few exceptions, cysteine residues present in potato sequences determine S-RNase’s tertiary structure. Sequence alignment studies have revealed the highly polymorphic nature of *S-RNases* among the Solanum species [37,41]. The *S-RNase* locus exhibits high allelic diversity, with amino acid similarity varying from 32.9% to 94.5% [37]. The observed polymorphism in S*-RNase* could be due to its ancient origin and diversification in the common ancestors of the Solanaceae [41]. All *S-RNase* genes identified to date belong to the class III type/group, as per the classification of plant T2-type RNases [14,42].

Interestingly, *S-RNases* of tuber-bearing species of potato show inter- rather than intraspecific similarities compared to 70 S-*RNases* of Solanum species, reflecting a case of trans-specific or trans-generic evolution of these S-alleles [41]. The interspecific nature of these similarities indicates that *S-RNase* is very old, has been inherited from a common ancestor, and has been passed on to different genera. Rosaceae family’s *Prunus* genus displays a similar pattern [43]. The observed S-locus polymorphism in angiosperms is attributed to the age of S-alleles, diversifying selection, and the tightly linked genes at the S-locus that preserve and maintain allelic variation at the S-locus [38,44]. The *Solanum* genus and other genera such as *Petunia* and *Nicotiana* display extensive S-allele diversification. Wild potatoes are diverse in terms of ploidy, ranging from diploid to hexaploid. Almost all the diploid potatoes are self-incompatible, whereas tetraploid potatoes are partially self-compatible and suffers from inbreeding depression. To date, *S-RNase* identified in potato genotypes are fewer in number when compared to other solanaceous crops, which demands further exploration of this gene family.

Another key gene governing SI in potatoes is the male determinant, *SLF/SFB*, which for many years went unrecognized. However, a detailed analysis of three plant families, the Solanaceae [11], Rosaceae [45], and Plantaginaceae [46], identified and provided evidence for the involvement of *SLF/SFB* in SI. It encodes the F-box protein involved in selecting targets for ubiquitination, in this case, S-RNase. However, there is uncertainty about SLF-mediated SI in the Solanaceae [47]. Moreover, the ubiquitination complex components appear differently between the Solanaceae and Plantaginaceae [48,49]. This could be one reason for the limited exploration of this gene family in potatoes. Detailed analysis of S-locus genes has provided a strategy to overcome the observed GSI in the diploid potato.

## 6. Molecular Approaches to Overcome SI

Based on the molecular mechanism of self-incompatibility in potatoes, two possible approaches could overcome the observed self-incompatibility in diploid potatoes (Figure 1). The first could be a manipulation of the S-locus, while the second could be the transfer of *Sli,* the locus used to overcome SI through conventional breeding. Within S-locus manipulation, there are two possibilities: the introduction of an extra *SLF* gene to degrade all S-RNases, by mutual weakening or by competitive interaction [16], and the knock-down of the *S-RNase* gene for its ribonuclease activity [36]. Apart from S-locus manipulation, another gene identified in *Nicotiana alata* designated as HT, which encodes a stylar-expressed small asparagine-rich protein, might overcome SI [50,51].

### 6.1. Inhibit the Function of the S-RNase Gene

The self-incompatibility mechanism is highly conserved within Solanaceae [8,51]. In the case of tomatoes, most wild accessions are self-incompatible. By contrast, the loss of function of *S-RNase* and other SI-related genes in the cultivated tomato have made it self-compatible, indicating that *S-RNase* is a prime target for manipulation to overcome self-incompatibility [36,51]. This natural occurrence in tomatoes inspired the idea of inhibiting the function of *S-RNase* in the diploid potato, thus overcoming observed self-incompatibility [36]. CRISPR-Cas9 now facilitates functional knockouts of *S-RNase*, which can overcome SI. As discussed earlier, the conserved domains in the *S-RNase* facilitate simultaneous knockout of S_p3_ and S_p4_ genes using a small guide RNA to target these conserved domains. Being codominant, the allelic mutation would enable pollen containing the same S-haplotype to fertilize the egg, resulting in self-compatibility. These loss-of-function events in *S-RNase* have successfully converted self-incompatible diploids to self-compatible ones, with growth vigor and plant morphology similar to the wild type [36]. The seed set in each berry varied from 67 to 288 seeds per fruit for each mutant line, sufficient to produce the next generation. The mutation in *S-RNase* is heritable and expected to perform a similar function in subsequent generations [36]. This potential indicates the prospect of using these mutant lines in breeding programs. SI in the *S. tuberosum* group Phureja clones (S15-47 and S15-76), *S. tuberosum* group Stenotomum clones (S15-48 and S15-107), *S. pennellii, S. habrochaites*, and *S. arcanum* has been overcome by a similar strategy [34].

### 6.2. Introduction of SLF/SFB

There is the possibility to overcome SI by introducing an extra *SLF/SFB* gene. Such an addition of extra *SLF/SFB* to the SI genotypes should degrade all S-RNases, either by mutual weakening or by competitive interaction [16]; however, this approach has gained limited success. This could become the most favored approach in the future because it is now known that *Sli* also encodes for products similar to *SLF/SFB* and functions similarly to them in eliciting the SC/SI response.

### 6.3. HT Knockouts

In *S. chacoense*, the stylar portion of the flower expresses small proteins named ScHT-A and ScHT-B. They are developmentally regulated during anthesis identically to the S-RNases following compatible and incompatible pollination. A gene silencing approach has elucidated the role of these isoforms in conferring self-incompatibility. This work has revealed that only the HT-B isoform is directly involved in self-incompatibility [52]. Thus, there is a need to create a similar self-compatible clone by exploiting the genes other than the S-locus. These kinds of efforts will generate genetically diverse clones for future exploitation in diploid breeding programs to develop RILs, NILs, mutant libraries, and introgression lines. Apart from these genetic engineering approaches, a more successful approach to overcoming SI is through introgression of *Sli* through conventional breeding [2].

### 6.4. S-Locus Inhibitor Introgression

Most diploids are SI; however, natural self-compatible mutants appear in several self-incompatible relatives [20]. The few SC clones are *S. chacoense* clones, chc 525-3 [23] and M6 (originally, chc 523-3) [2], *Solanum tuberosum* diploid RH89-039-16 [25], two dihaploids, US-W4 [26] and G254 [27] and *S. stenotomum* landrace Huasa Amarilla (a.k.a. C151 or CIP 705468) [24]. Dominant *Sli* identified from the wild potato species *Solanum chacoense*, chc 525-3 [23,53] has given rise to the possibility of converting SI to SC. It is necessary to understand the source of incompatibility in these natural mutants before using them to overcome SI. Before the *Sli* gene was identified, self-compatibility in these clones was attributed to the distal part of chromosome 12, evident from five independent mapping studies from these self-compatible clones. The first study was in *S. chacoense* clone chc 525-3, wherein *Sli* associated with the self-compatibility of this clone was mapped to the distal part of chromosome 12 [53]. In a second study, self-compatibility in the diploid *S. tuberosum* clone was again associated with that portion of the chromosome and was evident from the seed set after pollination [25]. A third study mapped self-compatibility QTL at the distal part of chromosome 12 in clone CD-320-20, derived from the dihaploid population of US-W4 [54]. The fourth study identified haplotype-specific SNPs linked to self-compatibility in two diploid mapping populations using comparative subsequence sets analysis (CoSSA). It further mapped this self-compatibility haplotype to the distal end of chromosome 12 using density graphs of unique k-mers mapped in 1 Mb bins of the reference genome DM v4.03 [30]. These results are consistent with earlier reports of the chromosome location of the *Sli* locus and its role in self-fertility [25,53].

Consistent with previous reports, one common haplotype involved in SC was mapped at a distal locus on chromosome 12. Further downstream analysis of a pool of cultivated tetraploid, landrace, diploid, and well-known SC clones has identified the candidate region for the *Sli* gene of 333 kb spanning recombination breakpoints at 58.945 Mb and 59.278 Mb [30]. CoSSA and the availability of an extensive data set generated by Hardigan et al. [55] paved the way for allele mining for *Sli* and fine-mapping of this locus. Using the nucleotide archive of clone M6, identifying this recombinant haplotype has narrowed the *Sli* locus’s candidate region from 1.5 Mb to 333 kb.

Haplotype-specific k-mers obtained with CoSSA can easily be converted into SC-associated competitive allele-specific PCR (KASP)™, which has proven instrumental in identifying the *Sli* gene. These markers facilitated the transfer and selection of SC in QTL mapping. Earlier, SC had been selected based on the previously discussed phenotyping methods. These phenotyping methods were destructive, time-consuming, and the data subject to quantitative variation, which led to ambiguity between self-compatibility and self-fertility. These marker systems may permit the transfer of SC to suitable SI breeding stock and the selection of SC progenies during the early stages of the diploid breeding program. Selfing of these progenies generally leads to 100% SC offspring because only *Sli*-containing pollen tubes reach the ovary and produce offspring, resulting in a rapid fixation of *Sli* in a homozygous state. KASP for *Sli* is highly reliable and is diagnostic of SC based on the genotype and phenotype. Putative *Sli* homozygous clones can be identified based on this marker system as part of marker-assisted breeding. The clones produce only SC offspring, demonstrating the reliability of these marker systems [37]. Clone M6 is widely used as donor for introgression SC. It has been found to be homozygous for six DNA KASP markers (Sli_090, Sli_561, Sli_304, Sli_626, Sli_898, and Sli_424), linked to *Sli* in Dutch germplasm and spanning a 224 kb region [29].

Previous reports indicate a recessive lethal allele linked to *Sli* based on the distorted segregation ratio of various SC plants [23,25,56,57,58,59]. This lethal allele has mapped to the distal end of chromosome 12, positioned about 1 Mb south of the *Sli* candidate region [30]. Based on KASP assays, heterozygosity at the *Sli* locus has not been observed in SC plants. A similar observation was found on the distal end of chromosome 12 of clone M6, which suggests the possibility that the lethal allele has recombined from *Sli* and does not limit homozygosity [30,60].

**Figure 2:**
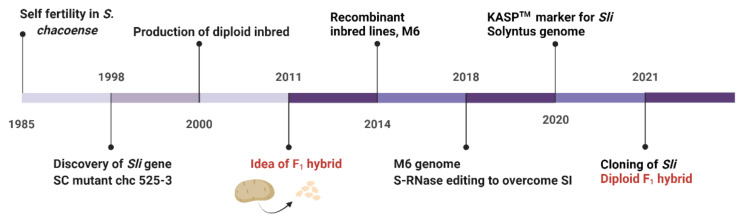
The major events in *Sli* gene discovery and development of F_1_ hybrid in diploid potato. Major events before the idea of F_1_ hybrid was conceived includes discovery of *Sli* gene in natural SC mutants of *S. chacoense* followed by development of inbred lines in diploid potatoes [1,53,61,62]. After that there have been major achievements like development of recombinant inbred lines, development of self compatible clone, M6 and *S-RNase* edited lines [2,36], sequencing of SC clones, M6 and Solyntus [32,63], development of DNA markers [30] and cloning *Sli* [33,34]. These important events contributed to the development of diploid F_1_ hybrid in potato [64].

The complicated and time-consuming SC phenotyping process and lack of sufficient SC clones have made it challenging to identify the molecular mechanism of the *Sli* gene. However, advancements in SNP genotyping using KASP marker technology, SC phenotyping methods, the availability of SC clone genome sequence information and precise genome editing tools like CRISPR-Cas9 have ended the hunt for the *Sli* gene in diploid potato. Figure 2 highlights the major events in *Sli* gene discovery and its identification.

## 7. *Sli* Gene Identification and Its Function

Two groups have independently hypothesized that *Sli* acts gametophytically. If true, this could result in segregation distortion in the progeny. Ultimately, PGSC0003DMG400016861 was identified as the candidate gene for *Sli* in different self-compatible diploid potato genotypes [33,34]. SNP markers proved highly instrumental in fine-mapping the interval spanning the *Sli* region that resulted in the identification of the candidate gene. The observed segregation distortion for SC in the F_2_ population of a cross between SI and SC has proven the gametophytic expression of *Sli.* Eggers et al. [33] used a recombinant screening approach to fine map the *Sli* interval from 628 kb to 12.6 kb. This effort ultimately led to the identification of the two genes PGSC0003DMG400016861 and PGSC0003DMG400016860 in *S. chacoense* (DS)-derived inbred lines [33]. Using a similar approach, Ma et al. [34] also mapped the SC loci in RH, another self-compatible long-y adaptive line derived from *S. tuberosum* group Tuberosum [34]. Its selection was based on recent mapping studies wherein *Sli* SC haplotypes were mapped at the distal end of chromosome 12 [30]. The observed segregation distortion (~1:1 segregation ratio of SC to SI) for self-compatibility in the F_1_ generation of a cross between RH (SC) and PI 225689 (SI), a self-incompatible diploid line (a/a) from *S. tuberosum* group Phureja, revealed *Sli* to be a single dominant heterozygous gene (A/a) or gametophytic factor [34]. Further, bulked segregant analysis (BSA) of 40 SC and 40 SI F_1_ individuals mapped the region for SC at the end of chromosome 12 (58~61 Mb); further, fine-mapping using InDel markers located the candidate within the interval between the markers M-1 and M-2. Furthermore, extreme segregation distortion (SD) in the F_2_ population and S_1_ progeny of RH suggested the gametophytic expression of *Sli.* Thus, only the pollen harboring the SC gene could penetrate the self-style and complete fertilization to produce progeny. All F_2_ progeny would be expected to carry the SC gene and exhibit SC phenotypic traits [34]. These two studies [33,34] have identified the two annotated genes, PGSC0003DMG400016861 and PGSC0003DMG400016860, in the interval mapped for SC on chromosome 12 in different SC genotypes. However, it was challenging to identify which of these two was the candidate gene; sequence variation analysis for these two genes in several whole-genome sequenced diploid potato lines identified all SC-specific SNPs and INDELS. Between these two, PGSC0003DMG400016861 shows six SC-specific amino acid substitutions and, notably, a 533 bp insertion located 108 bp from the start codon, suggesting that the SC allele has altered expression compared to the SI allele. Detailed molecular analysis showed a 533 bp insertion at 108 bp from the start codon as originating from a transposable element (TE) [33]. Furthermore, PGSC0003DMG400016861 specifically expresses in the pollen, which was evident from the transcriptome analysis of in-vitro germinated pollen from 10 SC and SI genotypes [33]. This large insertion at the promoter of *Sli* is common in all three self-compatible lines (RH, M6, and E172). In addition to these insertions, several point mutations within this region in self-compatible and self-incompatible lines may play an important role. However, this 533 bp fragment is speculated to be responsible for the pollen-specific expression of the *Sli* in SC lines. Testing this hypothesis would provide insights into the regulatory mechanism and the evolutionary trajectory of the *Sli* gene [34].

Further, the transcript of the *Sli* gene gradually increases with pollen development, reaching its maximum expression in the mature sporoderm [34]. Thus, the 533 bp insertion in the gene’s promoter is indeed responsible for this pollen-specific expression. Bioinformatic analysis revealed that the 533 bp insertion in the promoter originated from transposable elements, most probably from the MITE family [33]. Transposon insertions generally alter gene expression, including that reported earlier in potatoes, indicating that 533 bp insertion in its promoter causes the altered pollen-specific expression of *Sli.*

*Sli* identified from the RH genome has colinear regions at the distal end of chromosome 12 in other commonly used SC clones such as M6 and E172. When the gene sequences of these three genomes are compared, no sequence variation is evident in the promoter elements; however, five base pair differences resulting in two amino acid changes (C214R and Q249R) were evident in M6 [34].

To date, *Sli* has overcome SI in conventional breeding. The transgenic approach to overcome SI did not become a reality until the greater availability of sequence information of potatoes. However, the availability of *Sli* gene sequences has made it far easier to overcome SI with a transgenic approach. When SI diploid potato is transformed with an expression construct containing the exons of the SC allele of *Sli* between its native promoter and terminator, it succeeds in overcoming SI. Self-berry, seed set, and pollen tube penetration deeper into the style after self-fertilization provided shreds of evidence for the loss of SI. If *Sli* is the key player in SC, then its functional deactivation may lead to the loss of SC. Thus, editing the first exon of *Sli* in SC with CRISPR–Cas9 led to the loss of SC and resulted in SI progeny, providing further evidence of the exclusive role of *Sli* in overcoming SI in potatoes.

The name given to *Sli* upon its identification indicates the namer’s assumption that *Sli* somehow inhibited the S-locus [23]. However, it is now clear that its product is similar to that of another active gene in the S-locus, *SLF*. *Sli* may function as *SLF* to interact with and detoxify *S-RNases* during pollen tube elongation, thus conferring SC to self-incompatible lines [34]. It encodes an F-box protein, PP2-B10, which consists of an F-box domain linked to a lectin domain. Lectin domains are known to interact with carbohydrates and may interact with glycosylated proteins like S-RNase. The motifs located downstream of the F-box domain can confer substrate specificity for ubiquitination. Yeast two-hybrid assays and firefly luciferase (LUC) complementation assays revealed that the C-terminal region (Phloem Protein 2 domain) of *Sli* interacted with most of the *StS-RNases*. Testing *Sli* interaction on 14 *StS-RNases* revealed that *Sli* interacted with most *StS-RNases* except *3*, *9*, *10*, and *13*. However, full-length *Sli* (F-box and PP2 domain) failed to interact with *StS-RNase 3*, *9*, *11*, *13*, and *14* [34]. By contrast, the C-terminal region of a male *SLF* gene from RHC01H2G1617 could only interact with *StS-RNase9*, providing evidence that one SLF generally interacts with limited types of non-self *S-RNases*, as the collaborative non-self-recognition model predicts. To detoxify non-self *S-RNases*, and to out-cross, solanaceous plants must have multiple types of SLFs per S-haplotype [16].

Interaction studies have shown that various *StS-RNases* and *Sli* interact, providing a molecular basis for the loss of SI in diploid potatoes [34]. In addition, the collaborative non-self-recognition model of SI also remains conserved in Solanaceae [16]. It is interesting to note that although *Sli* codes for the F-box protein and behaves similarly to *SLFs*, it is not encoded by the S-locus; hence its naming as non-*S-locus* F box protein (NSF) [34]. Therefore, it is interesting to study the evolution of this gene in self-compatible diploid clones. 

It was suggested earlier, and in compliance with the two-step model [65], that *Sli* had evolved as a novel SLF. *Sli* was shown to interact with self S-RNase and with multiple types of the S-RNases for out-crossing with other diploids. Hence, the presence of *Sli* may represent an efficient route to introduce a fixed SC phenotype into S-RNase-based self-incompatible plants. However, *Sli* cannot interact with all types of *S-RNases*, indicating that the *Sli* gene cannot confer SC to all self-incompatible lines. Moreover, deleterious mutations linked to *Sli* also hamper the effort to overcome SI using this gene. Hence, using SC genes from different sources in breeding can effectively avoid these genetic bottlenecks caused by the deleterious mutations linked to these loci. With the availability of SC genotyping using KASP^TM^ markers, large potato germplasms can be readily screened for new sources of SC. These markers have identified the SC candidate region in potatoes. Pedigree analysis has shown Rough purple chili as the source of *Sli* in all European and North American varieties [30]. However, it is challenging to distinguish the SC haplotype between historical *S. tuberosum* cultivars and *S. chacoense* clones. *Sli* in *S. tuberosum* may be another example of gene flow between wild and cultivated potato germplasm, as observed for several other genes [55]. However, it is difficult to comment on the direction and timeframe of a putative introgression of *Sli*. Rough purple chili, the source in modern cultivars of Chilean *S. tuberosum* cytoplasm, has been hypothesized to have hybridized with the wild. *S. chacoense* or a more recent hybridization may have occurred between self-compatible *S. tuberosum* dihaploids and the ancestor of M6 [30].

K-mer analysis followed by distance estimation using Mash software explains the *Sli* locus’s origin in *S. chaocense* clone M6. This analysis has provided proof for the widespread occurrence of *Sli*, which may allow using many clones to breed SC diploid potato. It is proposed that SC can be introduced into *S. tuberosum* by crossing dihaploids from selected tetraploid varieties with M6 and other wild sources [24]. This analysis shows that the SC haplotype is widespread among the tetraploid varieties, and the one selected for dihaploid induction must contain *Sli*. Interestingly, KASP assays have found *Sli* in the dihaploids of potato cultivars ‘Atlantic’, ‘Superior’, and the breeding clone NY148 [29]. This shows that the diploid SC gene pool can be readily expanded without *Sli* introgression.

## 8. Developing Hybrid Potatoes

**Figure 3:**
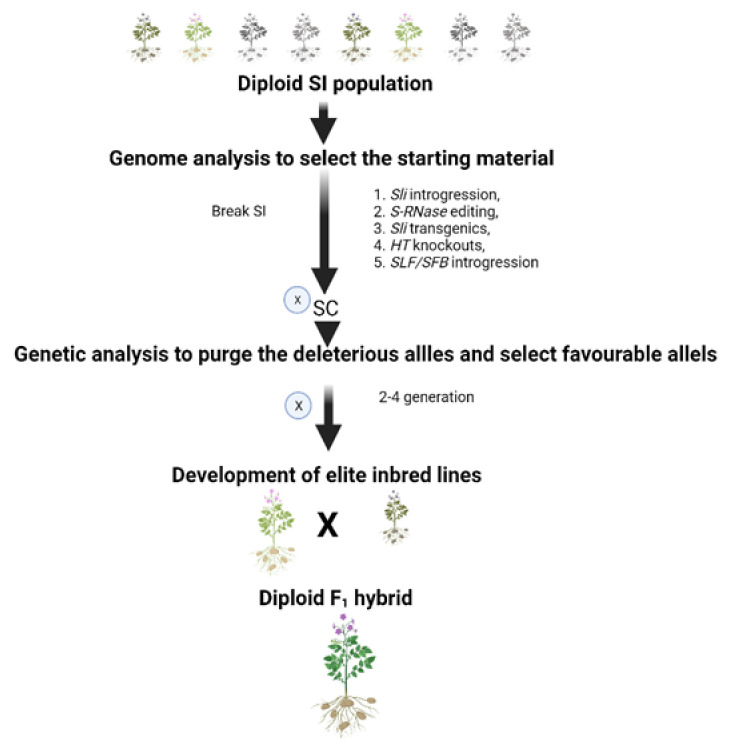
Genomic-assisted breeding approach for the development of a diploid F_1_ hybrid. Based on genome analysis, the starting material can be selected from the pool of diploid germplasm. The SI barrier of selected material can be overcome by five possible approaches: 1. S-locus inhibitor (*Sli*) is introgressed from a self-compatible *Sli* donor in which it naturally occurs; 2. Editing of S-RNase; 3. *Sli* gene transformation; 4. *HT* knockouts; or 5. *SLF/SFB* introgression. Elite inbred lines can be developed by selfing up to 2–4 generations after purging the deleterious alleles. The vigorous F_1_ hybrids can be developed by crossing the elite inbred lines from the different lineages.

Multiple approaches are now available to overcome the SI barrier in diploid potatoes, which allow selfing to generate an inbred line, the prerequisite to develop the F_1_ hybrid in potatoes. A great amount of diploid germplasm has been characterized for various traits and is available. This germplasm can be further used in diploid breeding programs to develop F_1_ hybrids. Most of them are SI, but now it is possible to transform them to SC using the approaches discussed above. To date, the conventional breeding approach to breaking SI has gained success [64]. But other approaches like *Sli* transgenics and *S-RNase* edited lines can be used to break SI. The lines can be phenotyped for compatibility using robust SC phenotyping methods. However, deleterious mutations in selfed populations pose great challenges to the development of highly homozygous inbred lines. Hence, it is essential to use starting materials with lower heterozygosity and fewer deleterious mutations. Deleterious mutations in starting material can be predicted based on amino acid conservation using the SIFT algorithm [31]. One potential challenge is to purge deleterious mutations from such lines. Genetic analysis can identify segregation distortion regions (SDs) and the deleterious and beneficial alleles in an S1 population. Highly homozygous inbred lines can then be developed by continuous selfing, and genome-assisted selection can be used to remove deleterious mutations and stack a beneficial allele. Crossing these elite lines from different lineages may permit the exploitation of heterosis in their hybrids [64]. A schematic representation of the development of a diploid F_1_ hybrid is shown in Figure 3.

## 9. Conclusions

Self-incompatibility has been the bottleneck in developing F_1_ hybrids in diploid potatoes. The advantages of hybrid TPS over conventional seed potato tubers resulted in considerable progress toward developing F_1_ hybrids in diploid potatoes in the last two decades. Identifying a natural mutant for SI in a *S. chacoense* clone has sown the seeds for this research. Now multiple approaches are available to overcome self-incompatibility in diploid potatoes. Among them, a conventional breeding approach has gained much success. The *Sli* donor has been used to overcome the SI in many diploid potatoes to generate the inbred lines. However, this approach is limited by the linkage drag associated with the *Sli* gene. Another hurdle in this direction was SC/SI phenotyping, but robust phenotyping methods have been developed over time.

Meanwhile, molecular markers have been developed for the *Sli* gene, which may play an important role in specific gene transfer by marker-assisted selection. With detailed molecular analysis of the SI mechanism in diploid potatoes, more rapid biotechnology options are available for editing the *S-RNAses* and *Sli* gene transfer using agrobacterium mediated transformation. These advances now make it possible to break SI and perform the selfing to generate inbred lines. Another potential challenge is to purge deleterious mutations and so avoid inbreeding depression, which can be achieved by genome-wide genetic analysis to identify segregation distortion regions (SDs) and the deleterious and beneficial alleles in S_1_ populations. However, the need remains to identify suitable diploid potato lines with comparable yield and suitability to a range of climatic conditions to that of tetraploid cultivars. A diploid F_1_ hybrid is in the embryonic stage but has the potential to transform the potato cultivation scenario as TPS will replace seed potato tubers for planting material. Adoption of TPS will reduce the cost of logistic support for storage, transportation, and management of tuber-borne diseases. The technology of potato hybridization at the diploid level is scientifically sound, technically feasible, economically viable, disease-free, and environmentally sustainable. TPS will provide smaller-scale farmers an opportunity to generate high-quality planting material and assure high yields of consistent-quality tubers with low inputs, compared to seed tuber of unknown health status.

## Figures and Tables

**Table 1:** Self-compatible clones used to date to explain self-compatibility in diploid potatoes.

Clone Name	Species	Ploidy ^1^	Remark	Reference
chc 525-3	*S. chacoense*	2*x*	*Sli* donor in Tuberosum	[23]
M6	S7 *S. chacoense*	2*x*	Male donor for introgression of SC, developed from chc 523-3	[2]
524–8	S7 *S. chacoense*	2*x*	Inbred *S. chacoense* clone from M6 breeding program	[2]
39-7	*S. chacoense*	2*x*	PI 275138	[28]
PI 133664–40	*S. chacoense*	2*x*	Genes segregate for SC in a 1:1 ratio	[29]
CIP 705468	*S. stenotomum*	2*x*	Landrace, Huasa Amarilla	[24]
US-W4	*S. tuberosum*	DH	Parthenogenetically produced from clone ‘20–20-34′	[30]
CD-320-20	*S. tuberosum*	DH	Clone derived from the US-W4	[30]
XD3	*S. tuberosum*	2*x*	Cross between US-W4 and 39–7	[28]
RH89-039-16 (RH)	*S. tuberosum*	2*x*	SC *Sli* haplotype at the distal end of chromosome	[25]
DMRH-89	*S. tuberosum*	2*x*	Cross between RH and *S. tuberosum* Group Phureja DM 1–3516 R44 (DM)	[25]
1S1	*S. tuberosum* (Phureja)			[29]
G254	*S. tuberosum*	DH	Dihaploid extracted from Gineke	[27]
IVP07-1001-4	*S. chacoense*	2*x*	*Sli/Sli*, WUR Plant Breeding	[23]
16HP01-66	*S. chacoense*	2*x*	*Sli/sli*, SC, Solynta breeding program	[31]
17SC25-8	*S. chacoense*	2*x*	*Sli/sli*, SC, Solynta breeding program	[31]
Solyntus	*S. chacoense*	2*x*	*Sli/Sli*, Solynta breeding program	[32]
16BL5033-2702	*S. chacoense*	2*x*	*Sli/Sli*, Solynta breeding program	[33]
18SC12-151	*S. chacoense*	2*x*	*Sli/sli*, SC, Solynta breeding program	
18SC11-19	*S. chacoense*	2*x*	*Sli/sli*, SC, Solynta breeding program	
17SC11-1157	*S. chacoense*	2*x*	*Sli/sli*, SC, Solynta breeding program	
17SC11-4016	*S. chacoense*	2*x*	*Sli/sli*, SC, Solynta breeding program	
320-02	*S. chacoense*		*Sli/sli*, USDA	
17SC100-18	*S. chacoense*	2*x*	*Sli/Sli*, Solynta breeding program	
17SC100-2	*S. chacoense*	2*x*	*Sli/Sli*, Solynta breeding program	
E172	*S. chacoense*	2*x*	Cross between SI, E and chc 525-3	[34]

^1^ 2*x* are diploids and DH are dihaploids.

## Data Availability

Not applicable.
